# Supplementary material for: On-Chip Antifouling Gel-Integrated Microelectrode Arrays for In Situ High-Resolution Quantification of the Nickel Fraction Available for Bio-Uptake in Natural Waters
Source: Molecules. 2023 Jan 31;28(3):1346. doi: 10.3390/molecules28031346 (PMC9919566; doi:10.3390/molecules28031346)
Supplement: Supplementary file 1 [file molecules-28-01346-s001.zip › molecules-2137706-supplementary.pdf]

Supplementary Materials

On-chip antifouling gel-integrated microelectrode arrays for in situ high-resolution quantification of Nickel fraction available for bio-uptake in natural waters

Sébastien Creffield[1], Mary-Lou Tercier-Waeber[1*], Tanguy Gressard[1], Eric Bakker[1], Nicolas Layglon[1*]

[1]University of Geneva, Sciences II, 30 Quai E.-Ansermet, 1221 Geneva 4, Switzerland

[*] Corresponding authors: marie-louise.tercier@unige.ch
nicolas.layglon@unige.ch

S.1. Field test area

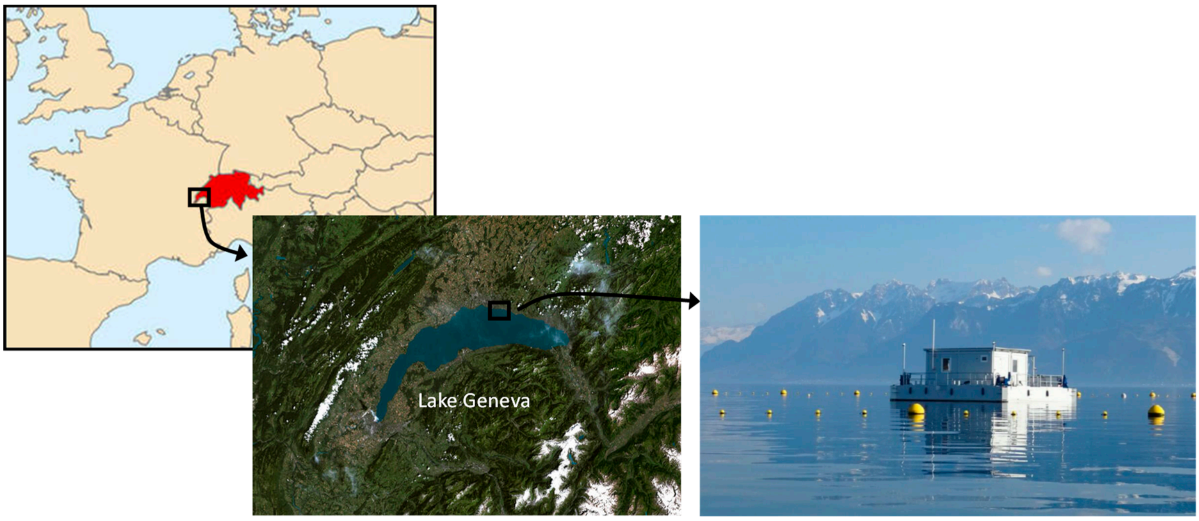

Figure S1: Map of the Lake Geneva associated to a picture of the LeXPLORE platform.

S.2. Ad-SWCSV measurements of the dynamic Ni-nioxime fraction

Table S1 : Summary of the Ad-SWCSV protocol for the determination of the dynamic Ni- and Co-nioxime fractions

**External cell Ni and Co Ad-SWCSV protocol**	**Flow-through cell Ni and Co Ad-SWCSV protocol**
Gel equilibration: 300 s<br>SW stripping and background parameters:<br>- E initial: -700 mV<br>- E final: -1300 mV<br>- Pulse amplitude: 25 mV<br>- Step amplitude: 4 mV<br>- Frequency: 200 Hz<br>CELL: On<br>Cleaning: E = -1200 mV; t = 30s<br>Preconc.: E = -700 mV; t = 90s<br>Equilibration: E = -700 mV; t = 10s<br>Stripping measurement<br>Cleaning: E = -1200 mV; t = 30s<br>Equilibration: E = -700 mV; t = 10s<br>Background measurement<br>CELL: OFF<br>Data storage (internal memory) and transfer (computer)<br>SW stripping and background parameters:<br>- E initial: -1200 mV<br>- E final: -200 mV<br>- Pulse amplitude: 25 mV<br>- Step amplitude: 8 mV<br>- Frequency: 200 Hz<br>CELL: On<br>Cleaning: E = -100 mV; t = 60s<br>Preconc.: E = -1200 mV; t = 120s<br>Equilibration: E = -1200 mV; t = 60s<br>Stripping measurement<br>Cleaning: E = -100 mV; t = 60s<br>Equilibration: E = -1200 mV; t = 60s<br>Background measurement<br>CELL: OFF<br>Data storage (internal memory) and transfer (computer)	Pump on: 180 s<br>Gel equilibration: 300 s<br>Pump on: 30s<br>SW stripping and background parameters:<br>- E initial: -700 mV<br>- E final: -1300 mV<br>- Pulse amplitude: 25 mV<br>- Step amplitude: 4 mV<br>- Frequency: 200 Hz<br>CELL: On<br>Cleaning: E = -1200 mV; t = 30 s<br>Preconc.: E = -700 mV; t = 90s<br>Equilibration: E = -700 mV; t = 10s<br>Stripping measurement<br>Cleaning: E = -1150 mV; t = 30s<br>Equilibration: E = -700 mV; t = 10s<br>Background measurement<br>CELL: OFF<br>Data storage (internal memory) and transfer (computer)<br>SW stripping and background parameters:<br>- E initial: -1200 mV<br>- E final: -200 mV<br>- Pulse amplitude: 25 mV<br>- Step amplitude: 8 mV<br>- Frequency: 200 Hz<br>CELL: On<br>Cleaning: E = -100 mV; t = 60s<br>Preconc.: E = -1200 mV; t = 120s<br>Equilibration: E = -1200 mV; t = 60s<br>Stripping measurement<br>Cleaning: E = -100 mV; t = 60s<br>Equilibration: E = -1200 mV; t = 60s<br>Background measurement<br>CELL: OFF<br>Data storage (internal memory) and transfer (computer)
